# Intravenous Paracetamol in Adjunct to Intravenous Ketoprofen for Postoperative Pain in Children Undergoing General Surgery: A Double-Blinded Randomized Study

**DOI:** 10.3390/medicina55040086

**Published:** 2019-04-01

**Authors:** Danguolė Rugytė, Jūratė Gudaitytė

**Affiliations:** Department of Anesthesiology, Lithuanian University of Health Sciences, 44307 Kaunas, Lithuania; jurate.gudaityte@kaunoklinikos.lt

**Keywords:** pain, postoperative, child, non-opioid analgesics, recovery of function, patient satisfaction

## Abstract

*Background and objectives:* The combination of non-steroidal anti-inflammatory drugs and paracetamol is widely used for pediatric postoperative pain management, although the evidence of superiority of a combination over either drug alone is insufficient. We aimed to find out if intravenous (i.v.) paracetamol in a dose of 60 mg kg^−1^ 24 h^−1^, given in addition to i.v. ketoprofen (4.5 mg kg^−1^ 24 h^−1^), improves analgesia, physical recovery, and satisfaction with postoperative well-being in children and adolescents following moderate and major general surgery. *Materials and Methods:* Fifty-four patients were randomized to receive either i.v. paracetamol or normal saline as a placebo in adjunct to i.v. ketoprofen. For rescue analgesia in patients after moderate surgery, i.v. tramadol (2 mg kg^−1^ up two doses in 24 h), and for children after major surgery, i.v. morphine-patient-controlled analgesia (PCA) were available. The main outcome measure was the amount of opioid consumed during the first 24 h after surgery. Pain level at 1 and over 24 h, time until the resumption of normal oral fluid intake, spontaneous urination after surgery, and satisfaction with postoperative well-being were also assessed. *Results:* Fifty-one patients (26 in the placebo group and 25 in the paracetamol group) were studied. There was no difference in required rescue tramadol doses (*n* = 11 in each group) or 24-h morphine consumption (mean difference (95% CI): 0.06 (–0.17; 0.29) or pain scores between placebo and paracetamol groups. In patients given morphine-PCA, time to normal fluid intake was faster in the paracetamol than the placebo subgroup: median difference (95% CI): 7.5 (1.3; 13.7) h, *p* = 0.02. Parental satisfaction score was higher in the paracetamol than the placebo group (mean difference: –1.3 (–2.5; –0.06), *p* = 0.04). *Conclusions:* There were no obvious benefits to opioid requirement or analgesia of adding regular intravenous paracetamol to intravenous ketoprofen in used doses. However, intravenous paracetamol may contribute to faster recovery of normal functions and higher satisfaction with postoperative well-being.

## 1. Introduction

Non-steroidal anti-inflammatory drugs (NSAIDs) as a part of multimodal treatment were shown to be effective analgesics for postoperative pain management in children. They reduce opioid requirement and opioid-related nausea/vomiting and may also improve analgesia. Therefore, NSAIDs are currently recommended for postoperative pain management in children without contraindications [1]. Intravenous ketoprofen, a propionic acid derivate, is one of the most widely used NSAIDs in children [2] and is also one of the currently recommended intravenous NSAIDs for pediatric postoperative pain management by the European Society for Paediatric Anaesthesiology [3].

Paracetamol, a low molecular weight phenol compound, is another analgesic widely used for pediatric postoperative pain. Its efficacy depends on the route and dose of administration. Benefits are greater when intravenous (i.v.) paracetamol in contrast to rectal forms is used [4,5]. Previous studies, including mainly adult patients, have shown that addition of paracetamol to an NSAID results in improved analgesia in 64% of studies, depending on the NSAID used and the type of surgery [6]. Despite very low evidence of improved outcomes in children [4], a combination of NSAID and paracetamol is a widely used practice. Recently, however, the concern regarding the safety of paracetamol when used in young age has been raised due to the association with the development of increased bronchial reactivity [7]. Thus, in pediatric populations the benefits of adding i.v. paracetamol to NSAID should outweigh the risks and also be cost-effective [8].

We have previously investigated the effects of intravenous ketoprofen for postoperative pain management in children and adolescents [9]. The advantages of adding regular 24-h intravenous paracetamol to intravenous ketoprofen in a pediatric population were not explored. Therefore, the primary aim of this study was to find out if i.v. paracetamol in a dose of 60 mg kg^−1^ 24 h^−1^, given in addition to i.v. ketoprofen (4.5 mg kg^−1^ 24 h^−1^), reduces opioid requirement and improves analgesia in children and adolescents following moderate and major general surgery. As pain is a subjective experience, our secondary aim was to find out if additional i.v. paracetamol is associated with the improved physical recovery. The final aim was to assess parents’, and whenever possible, patients’ satisfaction with the postoperative wellbeing at the end of the 24-h follow-up. In the present study, patients were randomized to receive either intravenous paracetamol or normal saline as a placebo in adjunct to intravenous ketoprofen.

## 2. Materials and Methods

The study was conducted in accordance with the Declaration of Helsinki. The institutional ethical approval was obtained on 05 October 2012 (protocol No. BEC-MF-10), and permission to conduct the study was issued by Kaunas Regional Biomedical Research Ethics Committee (No. BE-2-10) (International trial registration No. NCT02248493 at ClinicalTrials.gov).

The American Society of Anesthesiologists 1 or 2 class patients aged 1–18 years undergoing thoracic, orthopedic, genitourinary or plastic surgery with expected moderate to severe pain at least for 24 hours postoperatively were enrolled after their parents/caregivers gave written consent. Children who had a known allergy to paracetamol, tramadol, morphine, ketoprofen or other NSAIDs, diagnosed with oncology or central nervous system disease, renal or hepatic dysfunction, bronchial asthma, peptic ulcer, hemorrhagic diathesis, or under current treatment with NSAIDs or paracetamol, opioids or anticoagulants were excluded.

In this double-blinded placebo-controlled parallel group study, 54 patients were enrolled and assigned according to a computer-generated randomization sequence into one of the two treatment groups using a sealed, opaque envelope method. Children in the paracetamol group received paracetamol (Perfalgan®, Bristol Myers Squibb, France) 20 mg kg^−1^ intravenously (1% 2 mL kg^−1^, but no more than 100 mL or 1 g) at skin closure, 6 and 20 h thereafter. Children in the placebo group received 2 mL kg^−1^ (but no more than 100 mL) of normal saline (0.9% sodium chloride) at skin closure and 6 and 20 h thereafter. A nurse not otherwise participating in the study prepared the vials with identical appearance. All children received ketoprofen 1.5 mg kg^−1^ i.v. at skin closure, 8 and 16 h thereafter. Rescue analgesia was applied according to the severity of the surgical trauma and patients’ age. In children after minor to moderate surgery (further referred in the text as minor surgery: laparoscopic, arthroscopic, soft tissue, tendon and plastic surgery, minor and median bone osteotomies and osteosynthesis), i.v. tramadol 2 mg kg^−1^ up to two doses (maximum 4 mg kg^−1^) within 24 h was available. In 6- to 18-year-old children after major surgery (pelvic and renal surgery, surgery on thorax, thoracic cage, scapula or clavicula, bone elongation surgery, osteosynthesis of femur and/or tibiae), i.v. morphine-patient-controlled analgesia (PCA) was administered. The primary outcome measure was the total number of tramadol doses or the amount of morphine (mg kg^−1^) used over 24 h after surgery.

All patients were fasted overnight, and no clear fluids were allowed before surgery. Patients were premedicated with 2.5–7.5 mg oral (*n* = 43) or 2.5–5.0 mg i.v. (*n* = 11) midazolam. All children underwent standardized general anesthesia which was induced with propofol 4 mg kg^−1^ i.v. (*n* = 40) or sevoflurane by face mask *(n* = 14). Following i.v. fentanyl 1 µg kg^−1^, atracurium 0.5 mg kg^−1^ was given to facilitate intubation of the trachea. Anesthesia was maintained with sevoflurane up to 1 minimum alveolar concentration in oxygen and air. Additional doses of fentanyl 1–2 µg kg^−1^ were given when required. For i.v. fluid therapy, a lactated Ringer’s solution was given at the average rate of 10 mL kg^−1^ h^−1^. No regional blocks were used in any patient, and no patient received antiemetic prophylaxis.

After surgery, all patients were extubated and followed-up in the post-anesthesia recovery area. Pain intensity was assessed at 1 and every 8 h until 24 h after surgery. The ten-point visual color analogue scale (VAS), from the lower end of no pain to the upper end of the worst possible pain [10] in children aged 6–18 years old, and the 10-point face, legs, activity, cry, consolability (FLACC) [11] scale in children 1–5 years old were used. Pain score at 1 h and mean score over 24 h (mean of the scores at 8, 16, and 24 h) were the secondary outcome measures.

When the patients after moderate surgery were fully awake, pain intensity was assessed twice until 1 hour after surgery and extubation. Rescue tramadol 2 mg kg^−1^ was administered if the pain score was more than five. During the 24-h follow-up period, the second dose of 2 mg kg^−1^ of tramadol could be administered on patient request. No other opioid was available for these patients and no intravenous fluids were given postoperatively.

In children after major surgery (21 patients), rescue morphine-PCA was started after a loading dose of morphine 0.04 mg kg^−1^, which was given in the operating room following extubation of the trachea. If insufficient, additional boluses of 0.02 mg kg^−1^ could be administered according to the decision of the anesthetist. The PCA settings were as follows: bolus dose 0.02 mg kg^−1^, lock-out time 8 min, maximum 4-h dose 0.4 mg kg^−1^ and a background infusion 0.004 mg kg^−1^ h^−1^. Patients were instructed on how to use the PCA pump on the day of operation before pre-medication and surgery. No other opioid was available for these patients. Standardized postoperative infusion therapy with lactated Ringer’s solution to cover two-thirds of hourly physiologic maintenance demand based on the patient’s weight was administered for 24 h after surgery.

All patients after surgery were allowed to drink clear fluids according to their own preference with no time or volume restriction.

During the 24-h follow-up period, patients’ physical recovery was quantified by estimating the time to the resumption of normal oral fluid intake (without subsequent nausea/vomiting) and the first spontaneous urination after surgery. Nausea/vomiting (retching or expulsion of gastric contents), urinary retention (catheterization of the bladder) or any other side effects were noted as present or absent.

At the end of the 24-h follow-up period, parents, and when possible, patient satisfaction with overall well-being was assessed with a 10-point visual analogue scale from 0—totally unsatisfied—to 10—totally satisfied.

The participants, their parents/guardians, the anesthetist who administered anesthesia, the nurses, and the observer, who recorded the study parameters, were blinded to the patient’s group assignment.

### Statistical Methods

The sample size estimation was based on the results obtained in children treated with morphine-PCA and regular intravenous ketoprofen following major surgery [9]. Mean morphine consumption in these patients was 0.5 ± 0.2 mg. Assuming a 30% decrease [6] in morphine consumption with additional intravenous paracetamol and using a two-sided α-level of 0.05 and power of 0.90, 76 patients (38 in each group) should be included in the study.

Continuous variables were checked with a skewness and kurtosis test for normality of distribution. Abnormally distributed variables (patient characteristics, anesthetic and surgical variables) are presented as median (range) and compared with the Mann–Whitney test. Normally distributed variables (cumulative morphine consumption, pain scores, parent and patient satisfaction scores) are presented as the mean and 95% confidence intervals (95% CI) and compared with *t*-test for independent samples and mean differences (95% CI) calculated. Quantile regression analysis was used for the comparison of medians and difference with 95% CI calculated for the abnormally distributed time to oral intake and spontaneous urination after surgery. Nominal variables are expressed as number of cases (%) and compared with Chi-square test. Risk ratios (95% CI) are presented for differences in proportions where appropriate (side effects).

A *p*-value of less than 0.05 was considered statistically significant. All statistical analyses were performed using STATA 7 software (Stata Corporation, 4905 Lakeway Drive, College Station, Texas, 77845, USA).

## 3. Results

**Figure 1:**
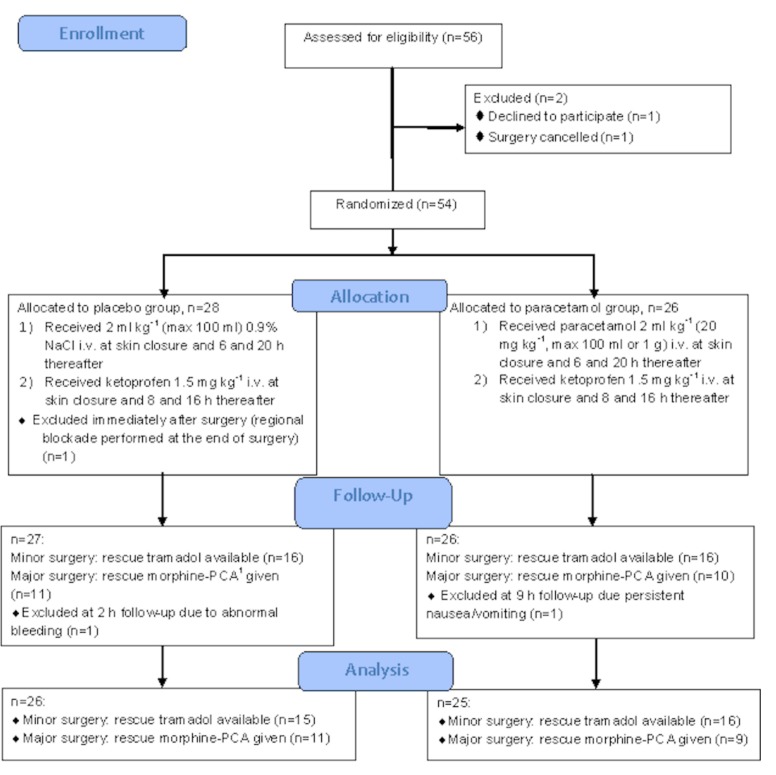
Flowchart of the studied patients. ^1^ PCA—patient-controlled analgesia.

Our aim was to include at least 76 patients. However, over the study period (November 2012–March 2017) regional blockade became a part of postoperative analgesia, thus we stopped the enrollment after 54 patients. There was one dropout from placebo group due to postoperative incision infiltration with local anesthetic by the surgeon. Thus, 53 patients were studied. One patient following plastic surgery was excluded at 2-h follow-up due to abnormal bleeding: the decision to stop subsequent doses of ketoprofen was taken. One patient following orthopedic surgery was excluded at 9-h follow-up, as rescue morphine-PCA was discontinued due to persistent nausea and vomiting. The flow diagram of the studied patients is shown in Figure 1.

The placebo and paracetamol groups did not differ according to patient demographic characteristics, anesthesia, surgery data, and the method of postoperative rescue analgesia (Table 1).

Opioid intake within 24 h after surgery did not differ between placebo and paracetamol groups (Table 2). In the subgroup of patients after minor surgery, with rescue tramadol available, only one patient required two doses of tramadol (placebo group), the rest were given a single dose. Median (range) time for tramadol administration was 40 (10–80) min with no difference between placebo and paracetamol groups (45 (10–80) versus 35 (10–65), respectively). In patients after major surgery treated with morphine-PCA, there was no difference in cumulative morphine consumption between placebo and paracetamol groups.

**Figure 2:**
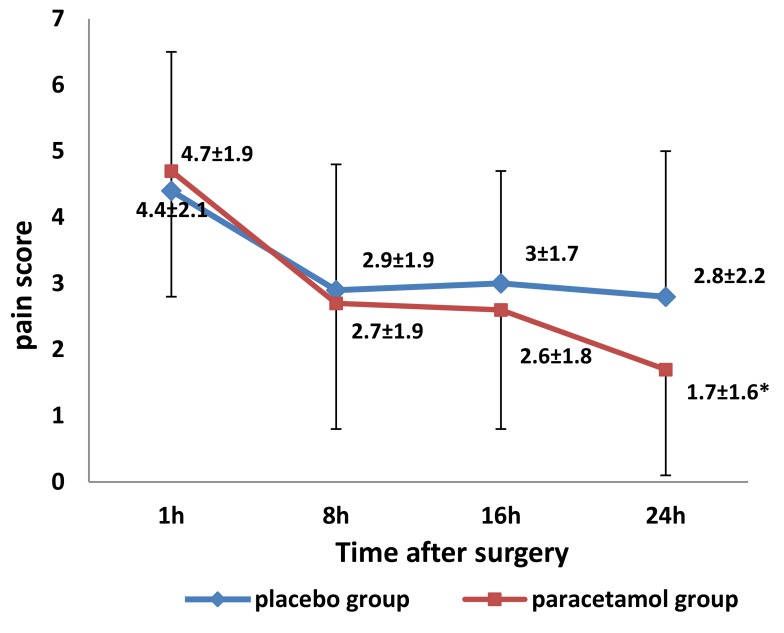
Mean (± standard deviation) pain scores 1 and every 8 h after surgery in the placebo and paracetamol groups (٭ *p* = 0.045, compared to the placebo group at 24 h after surgery, by *t*-test for independent samples at each time point).

Pain scores within 24 h after surgery are shown in Figure 2. Scores at 1 hour did not differ, and there was no statistically significant difference in mean pain scores over 24 hours between placebo and paracetamol groups (Table 2).

Time to the resumption of normal oral fluid intake after surgery was shorter in the paracetamol group in the subgroup of patients after major surgery, given rescue morphine-PCA (Table 3). No difference existed in patients after minor surgery with rescue tramadol available. In general, median (range) time to oral fluid intake was faster in patients without nausea/vomiting, compared to patients suffering this side effect (2.4 (0.5–10.0) h versus 7.0 (3.0–22.0) h, respectively, difference with 95% CI: 4.6 (1.1; 8.1), *p* = 0.012.

Time to the first spontaneous urination after surgery did not differ between placebo and paracetamol groups. Overall, mean time to resumption of both functions was shorter in paracetamol than placebo group (Table 3).

There was weak evidence for the lower incidence of nausea/vomiting in the paracetamol group, compared to the placebo (Table 4), with no statistically significant difference between the groups. There were no differences in the incidence of urinary retention and abnormal postoperative bleeding (managed conservatively) between the placebo or paracetamol groups (Table 4). Two patients in the paracetamol group complained of abdominal pain not related to surgery.

Parents/guardians rated overall satisfaction with the postoperative well-being higher in the paracetamol group than in the placebo group; however, the children themselves did not (Table 4).

## 4. Discussion

The main finding of our study was that regular 24-h administration of intravenous paracetamol in addition to a high-dose intravenous NSAID (i.e., ketoprofen) was not associated with decreased opioid intake, but was associated with benefits to the recovery profile of studied patients.

In the adult and pediatric population, the combination of paracetamol with different NSAIDs has been recommended and is being used worldwide for postoperative pain relief after major and minor abdominal, thoracic, urological, and orthopedic surgeries [12]. The Cochrane database recommendations advocate for the positive opioid-sparing and additive analgesic effects of combinations of paracetamol and NSAIDs in adults [13]. However, their use must be balanced against the risks of major adverse cardiac events associated with selective cyclooxygenase 2 inhibitors and gastrointestinal bleeding, nephrotoxic or hepatotoxic effects of other NSAIDs, especially in the aging population with polymorbidities and polymedication [14]. However, studies of postoperative multimodal analgesia have examined the use of a variety of different analgesics in a variety of procedures and report inconsistent outcomes, preventing a consensus on the superiority or risks of combining different classes of analgesics [15]. To overcome these shortages, a multicenter trial aiming to assess the benefits and risks of combined different doses of paracetamol and ibuprofen for adult patients after total hip arthroplastic surgery has been planned [16]. One of the major aims of the aforementioned study is to demonstrate that a combination of low doses of NSAIDs and paracetamol is equally effective but safer than high doses of each constituent alone. The results of this trial are still to come.

In contrast to adults, the great majority of pediatric patients lack comorbidities, which can lead to NSAID-related serious adverse effects. Based on the evidence of significant analgesic activity, NSAIDs are recommended for postoperative pain management in children without contraindications [1]. Few studies, though, compared a combination paracetamol-NSAID against NSAID alone in a pediatric postoperative setting. Based on the results of the two trials, included in a previously published meta-analysis, addition of paracetamol to an NSAID was associated with better pain scores over 24 h after surgery with no effect on opioid consumption [1]. One of these two studies was by Hiller A. et al. [17], which concluded that combination of rectal paracetamol and intravenous ketoprofen produced better analgesia than ketoprofen alone only in orthopedic, but not other types of surgery. However, like in our study, opioid requirement did not differ between combination and ketoprofen groups. Our population, though, was too small to draw conclusions regarding different types of surgery. A later systematic review in children [4], including three trials comparing a combination paracetamol-NSAID over NSAID alone, reported contradictory results: one trial in children after appendicectomy showed no effect on analgesia or opioid consumption of combination versus NSAID [18], another trial in children after cleft palate repair showed positive effect on analgesia, but not on opioid consumption [19], the third trial in children after adenoidectomy showed no effect in early postoperative period, but positive effect on analgesia after discharge home [20]. Yet, the other two trials, not included into the systematic review, again, reported opposite findings: decreased opioid requirement with single-dose pre-operative paracetamol–diclofenac combination over diclofenac alone was reported in children following inguinal hernia repair [21], whereas the study including children undergoing adenotonsillectomy showed no additional value on analgesia of a combination paracetamol–NSAID over NSAID alone during 48 hours after surgery [22]. The diversity in the results is explained by different pain models and different dosing schedules of paracetamol and NSAIDs. Among all the abovementioned trials, only one reported the effect of combination paracetamol and NSAID versus NSAID alone in children, treated with PCA-morphine postoperatively: no additional effect of rectal paracetamol on opioid requirement or pain scores was seen in children given rectal diclofenac-PCA-morphine combination after appendicectomy [18].

In contrast to our present study, all previously published studies used enteric, but not intravenous formulations of paracetamol. Intravenous paracetamol may result in faster and higher serum concentrations compared to rectal or oral routes [23]. Thus, stronger effect on pain alleviation would be expected. However, we were unable to demonstrate either differences in pain scores or opioid requirement with the addition of intravenous paracetamol to intravenous NSAID. One of the reasons may be that paracetamol was added to a high-dose NSAID (ketoprofen 4.5 mg kg^−1^ 24 h^−1^)—it was suggested that the analgesic benefits of additional paracetamol may become evident with low, but not high doses of NSAIDs (specifically, ibuprofen) [24]. Yet, another reason may be that we used sequential instead of simultaneous administration of paracetamol and ketoprofen. It has been suggested that the simultaneous administration of enteral forms of paracetamol and NSAIDs (ibuprofen) may result in prolonged analgesia of 4–8 h [24]. However, no data exist on the combination of intravenous forms of these medications. We selected a sequential versus simultaneous administration regimen in order to reach hypothetically more stable plasma concentrations of analgesics instead of simultaneous peaks and gaps. This might have resulted in less evident additive effects of both medications. However, this assumption is speculative as there are no effect-related pharmacokinetic data describing sequential administration of paracetamol and an NSAID. It has also been demonstrated that higher single doses of paracetamol correlate with better and faster onset of analgesia [25]. Thus, we used 20 mg kg^−1^ of intravenous paracetamol three times per 24 h, instead of generally recommended 15 mg kg^−1^ every 6 h. High doses (30 mg kg^−1^ every 8 h) of intravenous paracetamol in children and adolescents undergoing major surgery were described without the evidence for toxicity [5].

Effective pain treatment is one of the important complex measures in enhanced recovery after surgery programs. Nevertheless, recovery of functions is seldom reported in pain management trials. Addition of paracetamol to an NSAID in our study was associated with faster oral fluid tolerance, particularly in patients given morphine-PCA. Faster oral intake with the combination of acetaminophen–ketorolac–opioids over opioids alone and opioids–acetaminophen was recently reported in an observational study of adolescents after scoliosis surgery [26]. However, no comparison was provided with the opioids–ketorolac group. Faster oral intake between patients given morphine-PCA in our study was probably due to fewer patients with nausea/vomiting in the paracetamol subgroup (2 out of 10 versus 6 out of 11 in the placebo subgroup) as patients, who did not vomit, were able to tolerate fluids faster. Former systematic review and meta-analysis showed that intravenous paracetamol (with or without NSAID) was associated with less nausea/vomiting following surgery [27]. Potential mechanisms include improved pain relief and/or direct antiemetic effect of paracetamol.

We found that parental satisfaction with their child’s postoperative well-being was higher in patients, given additional paracetamol, compared to ketoprofen only patients. Satisfaction with postoperative pain treatment was previously reported to be higher in children treated with combination acetaminophen–ketorolac compared to placebo after inguinal hernia repair [28]. However, no comparison was provided with the ketorolac only group. The patients’ satisfaction scores in our study, though, did not differ. It may be that children and their parents consider different things when reporting satisfaction with postoperative well-being.

The safety of administering analgesics to children is important. Therapy by combination of paracetamol and ibuprofen was shown not to cause increased risk of adverse effects than either drug alone in non-surgical adult patients [29]. The information on safety of the combination therapy in pediatric patients undergoing general or major surgery is insufficient. We observed that there were two patients in the paracetamol group with abdominal pain not related to surgery. Whether this could be attributed to the paracetamol–ketoprofen combination is unclear. We also observed abnormal bleeding in two children in the placebo group: both patients were managed conservatively (change of wound dressings) and did not require return to the operating room. No risk of clinically significant bleeding was found in adult surgical patients given NSAIDs for postoperative pain management [30]. There are no sufficient data regarding the safety of the NSAIDs and the risk of postoperative bleeding in children following major general surgery.

Recently, the concern, regarding the use of tramadol in children has been raised due to the underappreciated awareness of tramadol-associated respiratory depression, especially in children at risk [31]. Nevertheless, tramadol is one of the recommended analgesics for postoperative analgesia in children [3]. We used 2 mg kg^−1^ of tramadol as a rescue dose in children 1 year and over, based on previous recommendations. We assumed though that a lower or titrated dose of tramadol to the desired effect could have been a safer alternative. However, respiratory depression was not observed in any of our patients treated with tramadol.

## 5. Limitations

This was a double-blinded placebo-controlled randomized study; however, our patients were not homogenous with regard to the type of surgery, as well as to the method of rescue postoperative analgesia. We also did not include a paracetamol only group, as it would be in contrast to our standard clinical practice. We did not include patients undergoing abdominal surgery, as this would have made it impossible to assess the time to oral intake. This may diminish the generalization of our study results. Our study was underpowered to detect statistically significant differences in the rate of opioid-related nausea/vomiting; however, we provided some evidence that a combination of regular intravenous paracetamol and NSAID might be useful in children after major surgery. Due to a small sample size, further research is required in these patients. There are several other limitations. The time points for pain assessment were infrequent and close to subsequent ketoprofen dose. Thus, we cannot exclude the potential masking of the paracetamol effect. More frequent pain assessments could have been more sensitive to detect potential analgesic effects of intravenous paracetamol. Tramadol administration on patient/parent request beyond the recovery period is another possible cause of bias in quality of analgesia between paracetamol and placebo groups, as, probably, only the patients in moderate to severe pain would actively search for means to alleviate it. However, this approach is used in pain management trials [32]. In addition, good correlation exists between child’s and parent’s pain rankings [33]. We selected this approach as a marker of moderate to severe pain in the environment of limited human resources dedicated to research around 24 h. Postoperative fluid intake might have been biased by preoperative fasting, and intraoperative and postoperative infusion therapy. The later was standardized, whereas, intraoperative fluid therapy and circadian start of surgery were comparable between the two study groups. However, although all patients were fasted overnight, we cannot exclude that actual fasting hours were different between the placebo and paracetamol groups. Additionally, the fasting interval was not in line with the current recommendations, allowing children to take clear fluids up till 1–2 hours to anesthesia [34].

## 6. Conclusions and Clinical Implications

This study indicates that there are no obvious benefits to analgesia or opioid requirement of adding regular intravenous paracetamol to intravenous ketoprofen in used doses. However, intravenous paracetamol may contribute to faster physical recovery in children after major general surgery and higher parental satisfaction with their child’s well-being. Our study supports the notion that pain scores or opioid consumption per se may not be sufficient outcome measures in analgesic trials. When acceptable pain relief is achieved over the majority of the follow-up period, recovery of normal functions or patient satisfaction can be important. Thus, based on the results of this study, regular intravenous paracetamol as an adjunct to a high-dose intravenous NSAID is of limited value in pediatric patients, following minor to moderate general surgery. However, we would recommend a combination of intravenous paracetamol–NSAID in patients requiring continuous opioid techniques for severe postoperative pain treatment, as it may enhance physical recovery and improve satisfaction. Larger or multicenter trials assessing the efficacy and safety of different dosing schedules of intravenous paracetamol administered in addition to an NSAID in children undergoing major surgery are required.

## Figures and Tables

**Table 1:** Patient characteristics, anesthesia, surgery data, and method of postoperative rescue analgesia.

Variable	Placebo Group, *n* = 27 ^1^	Paracetamol Group, *n* = 26 ^2^
Age (years)	13 (1–17)	12 (1–17)
Age groups: 1–5/6–18 years (*n*)	3/24	3/23
Weight (kg)	52 (10–72)	52 (9–86)
Gender: female/male	13/14	14/12
Thoracic surgery (*n*)	4	5
Orthopedic surgery (*n*)	16	15
Genitourinary surgery (*n*)	3	3
Superficial and plastic surgery (*n*)	4	3
Duration of surgery (min)	105 (60–240)	110 (60–360)
Intraoperative fentanyl dose (µg kg^−1^)	4.2 (2.1–9.4)	3.95 (1.8–12.3)
Circadian start of surgery (h)	10 (9–13)	10 (9–13)
Minor surgery (rescue analgesia: tramadol)	16	16
Major surgery (rescue morphine-PCA^3^)	11	10
Loading dose of morphine (mg kg^−1^) in rescue PCA patients	0.048 (0.03–0.14)	0.040 (0.04–0.11)

Data are median (range) or number of cases. ^1^ One patient was subsequently excluded at 2 h follow-up. ^2^ One patient subsequently excluded at 9 h follow-up. ^3^ PCA—patient-controlled analgesia.

**Table 2:** Comparison of opioid intake and pain scores between placebo and paracetamol groups.

	Placebo Group, *n* = 26	Paracetamol Group, *n* = 25	Difference	*p*
**Opioid Intake**				
Tramadol doses/no of patients ^1^ in the minor surgery subgroup	11/15	11/16	-	0.56
Postoperative 24 h morphine consumption (mg kg^−1^) in the major surgery subgroup	0.38 (0.20; 0.56)	0.32 (0.15; 0.49)	0.06 (–0.17; 0.29)	0.60
**Pain Scores**				
Pain score at 1 h	4.4 (3.5; 5.3)	4.7 (3.9; 5.4)	–0.26 (–1.42; 0.90)	0.66
Mean pain score over 24 h	2.9 (2.2; 3.6)	2.3 (1.8; 2.9)	0.6 (–0.3; 1.5)	0.21

Data are mean (95% confidence intervals) or number of cases. ^1^ All, except one patient (in the placebo group), were given a single dose of tramadol.

**Table 3:** Comparison of the recovery of normal oral and urinary functions between the placebo and paracetamol groups.

	Placebo Group, *n* = 26	Paracetamol Group, *n* = 25	Difference	*p*
**Time to Oral Fluid Intake (h):**				
Minor surgery	2.4 (0.5–21.8)	2.6 (1–7)	–0.2 (–1.5; 1.1)	0.7
Major surgery	10 (3.7–22)	2.5 (0.5–10)	7.5 (1.3; 13.7)	0.02
**Time to Spontaneous Urination (h):**				
Minor surgery	5.43 (2.2–24)	4.1 (0.5–7.2)	1.5 (–4.0; 7.0)	0.58
Major surgery	7.3 (1.25–16)	6.35 (2.8–9)	0.6 (–6.9; 8.1)	0.87
**Overall** (mean time to resumption of both functions) **(h):**	6.35 (0.5–22.1)	3.6 (1–9)	3.0 (0.4; 5.6)	0.024

Data are presented as median (range). Differences are presented with 95% confidence intervals.

**Table 4:** Comparison of side effects and satisfaction rates between the placebo and paracetamol groups.

	Placebo Group, *n* = 26	Paracetamol Group, *n* = 25	Difference	*p*
**Side effects**				
Patients with nausea/vomiting	11/26 (42.3%)	6/26 ^1^ (23.1%)	1.83 (0.80; 4.22)	0.14
Urinary retention ^2^	1/25 (4%)	1/20 (5%)	0.8 (0.05; 12.00)	0.87
Bleeding	2/27 ^3^ (7.4%)	0/25	-	0.16
**Satisfaction**				
Parents (*n* = 32) ^4^	7.2 (6.3; 8.1), *n* = 18	8.5 (7.6; 9.4), *n* = 14	–1.3 (–2.5; –0.06)	0.04
Patients (*n* = 40) ^4^	7.1 (6.3; 8.0), *n* = 20	7.6 (6.7; 8.5), *n* = 20	–0.44 (–1.64; 0.77)	0.47

Data are presented as number of cases (%). Differences are presented as risk ratio (95% CI). ^1^ The patient who was withdrawn from the study at 9 h follow-up is included. ^2^ In patients without urinary catheters and without the two patients who were excluded at 2 and 9 h follow-up. ^3^ The patient who was withdrawn from the study at 2 h follow-up is included.^4^ Data are presented as mean (95% CI).
